# Efficacy and safety of scopolamine compared to placebo in individuals with bipolar disorder who are experiencing a depressive episode (SCOPE-BD): study protocol for a randomised double-blind placebo-controlled trial

**DOI:** 10.1186/s13063-022-06270-4

**Published:** 2022-04-23

**Authors:** Cerena Miravalles, Ruán Kane, Eimear McMahon, Colm McDonald, Dara M. Cannon, Brian Hallahan

**Affiliations:** 1 The Centre for Neuroimaging and Cognitive Genomics (NICOG), Clinical Neuroimaging Lab, NCBES Galway Neuroscience Centre, College of Medicine, Nursing, and Health Sciences, National University of Ireland Galway, Galway, Ireland; 2 Health Research Board – Clinical Research Facility Galway, National University of Ireland Galway, Galway, Ireland; 3 University Hospital Galway, Galway-Roscommon Mental Health Services, Galway, Ireland

**Keywords:** Bipolar disorder, Scopolamine, Depressive episode, Cholinergic, Muscarinic, Antidepressant

## Abstract

**Background:**

Current treatment options for the management of depressive episodes in bipolar disorder are often sub-optimal, with some treatments either noted to be only partially effective or to require long durations of treatment prior to a therapeutic response. Therefore, pharmaco-therapeutic options that reduce depressive symptoms in a more rapid manner might provide a viable therapeutic option for some people. Intravenous (IV) scopolamine, a pan muscarinic antagonist, has been demonstrated in a number of studies to confer a rapid antidepressant effect, albeit no study to date has exclusively evaluated its potential therapeutic effect in a cohort consisting solely of individuals with bipolar disorder.

**Methods:**

Individuals with bipolar disorder who are currently experiencing a depressive episode of at least moderate severity will be included in this study. Eligible participants will undergo a screening and placebo-run in visit and will be randomised at visit 3 to the treatment or placebo group. Participants will receive the three blinded infusions over the course of 2 weeks, with two subsequent follow-up visits, 1 and 3 weeks after the last infusion visit. The total duration of the study will be approximately 6 weeks. Patients will continue their regular treatment regime in addition to study medication. Objective and subjective mood questionnaires, cognitive assessments and other psychometric instruments will be administered and recorded.

**Discussion:**

To our knowledge, this is the first study to investigate the antidepressant effects of IV scopolamine in an exclusively bipolar disorder cohort. Trial findings will contribute to the evidence base regarding the cholinergic hypothesis of mood disorders and specifically might result in an additional safe therapeutic option for the management of depressive episodes in bipolar disorder.

**Trial registration:**

ClinicalTrials.gov NCT04211961. December 26, 2019. EudraCT Number 2017-003112-39

## Background

Bipolar disorder is a chronic disabling psychiatric disorder characterised by recurrent episodes of mania or hypomania and depression. Individuals with bipolar I disorder experience episodes of mania and depression, while individuals with bipolar II disorder experience episodes of depression with periods of hypomania, but not mania [1]. Bipolar disorder has an estimated prevalence of approximately 1% and a roughly equal gender ratio [2].

Current pharmacological treatments for depressive episodes in bipolar disorder remain sub-optimal, with pharmacological strategies employed to date often only partially effective [3, 4]. A number of recent studies have suggested that scopolamine, a pan muscarinic(M) receptor antagonist, can elicit a rapid antidepressant response in both major depressive disorder (MDD) and bipolar disorder [5–7] and thus may present a novel therapeutic strategy, particularly for the management of bipolar disorder, in individuals experiencing depressive episodes [8].

A rapid-acting antidepressant effect has been demonstrated in some double-blind placebo-controlled trials of intravenous (IV) scopolamine in MDD or bipolar disorder [6, 7, 9, 10], but these findings have not been universally reported with other trials not reporting a significant treatment effect [11, 12]. Some studies have also examined other modes of scopolamine administration. For example, a previous study of intramuscular scopolamine demonstrated no antidepressant effect [11]. A randomised controlled trial (RCT) of oral scopolamine as an augmentation agent demonstrated an antidepressant effect; however, this effect was not rapid-acting and the patient cohort did not include individuals with bipolar disorder [13]. RCTs relating to scopolamine administration utilising a transdermal patch for the management of depressive episodes have yet to be published. Thus, studies of IV scopolamine as a treatment agent for depressive episodes whilst promising in relation to their potential therapeutic effect have to date included limited numbers of participants and diagnostically and clinically heterogeneous populations. This study is consequently significant as it will be the first RCT to date, to exclusively examine depressive episodes in bipolar disorder to ascertain if a rapid antidepressant effect is demonstrated.

Scopolamine, also known as “hyoscine”, is most commonly utilised for the management of post-operative nausea, motion sickness and hypersalivation secondary to psychotropic medications such as clozapine [14–16]. Scopolamine acts as an inhibitor at postganglionic muscarinic cholinergic receptor sites in the parasympathetic nervous system. Muscarinic cholinergic receptors (CHRMs), which recognise the neurotransmitter, acetylcholine (ACh), are a family of seven-transmembrane domain receptors consisting of five receptor subtypes (M1–5) and are associated with heterotrimeric G-proteins which translate a transduction cascade [17, 18].

The cholinergic system has long been implicated as a contributory factor in the aetiology of mood disorders. Physostigmine, a cholinesterase inhibitor (opposite effect to scopolamine), has been demonstrated to exacerbate depressive symptomatology [19, 20] and rescue deficits in emotion inhibition [21] in individuals with bipolar disorder. These observations have led to the hypothesis that an imbalance between central cholinergic and adrenergic neurotransmitter activity could induce manic and depressive episodes [20]. Both, over-activity and oversensitivity of the cholinergic system have been associated with depressive symptomatology, with a reduction in M2 receptors in vivo noted during depressive episodes [22], which is partially explained by variation in the M2 gene [23]. M2-receptor antagonism by scopolamine may potentially regulate activity of this system and therefore reduce the oversensitivity associated with depressive episodes. Consequently, evidence of a putative rapid antidepressant effect with IV scopolamine infusions may be of particular benefit for individuals with bipolar disorder who are experiencing depressive episodes. IV scopolamine could thus present an additional pharmacological strategy for the management of clinical and cognitive signs, symptoms and deficits in bipolar disorder by alleviating depressive symptomatology and ameliorating patient functioning.

The aim of this clinical trial is to investigate the efficacy and safety of IV scopolamine, compared to placebo, in reducing the severity of depressive symptoms in individuals with bipolar disorder who are experiencing a depressive episode of at least moderate severity. In addition, the study will investigate if IV scopolamine impacts cognition and improves functioning as measured on a number of objective and subjective psychometric instruments when compared to placebo.

## Methods

### Study design

This is a single-site, randomised, double-blind, placebo-controlled, parallel, phase IIb clinical trial examining if IV scopolamine exhibits an antidepressant effect in individuals with bipolar disorder. Study visits will take place at the Clinical Research Facility Galway (CRFG), in University Hospital Galway, Galway, Ireland. Patients will be recruited from mental health services in the West of Ireland including a specialised bipolar disorder clinic at University Hospital Galway.

Individuals will have a diagnosis of bipolar disorder according to the Diagnostic Statistics Manual (DSM)-V and must be experiencing a depressive episode of at least moderate severity based on a clinical interview by a trained clinician and a Hamilton Depression Rating Scale (HDRS) score ≥ 14. Exclusion criteria include being euthymic or experiencing a manic or hypomanic episode, treatment with any cholinergic medications (i.e. biperiden, procyclidine), a history of a previous allergic reaction or sensitivity to scopolamine, treatment with oral steroids, a history of an alcohol or substance use disorder in the 3 months prior to study entry, an intellectual disability (intelligence quotient < 70), dementia or cognitive impairment, and severity of depressive episode so that participation in a clinical trial is not appropriate.

Patients meeting all inclusion criteria will be enrolled into the study (duration ~ 6 weeks total). The maximum time between visit 1 (screening) and visit 2 is 14 days. The screening visit and visit 2 can occur on the same day. At visit 2, all participants will receive a placebo run-in (100 ml of saline IV). This has been utilised in other IV scopolamine RCTs [5–7, 9] and has the advantage of reducing a subsequent placebo effect (which might result in a Type II error) and a lower loss to follow-up rate post-randomisation [19, 24].

Within 7 days of visit 2, participants will be assessed against the placebo run-in criteria and if these criteria are met (HDRS score ≥ 8 at visit 3), they will be randomised to receive either placebo (*n* = 25) or 4 μg/kg scopolamine (*n* = 25) IV in 100 ml of saline over 15 min at:

- Visit 3 (Day 0),
- Visit 4 (days 2-6) and
- Visit 5 (days 6-10)

**Fig. 1:**
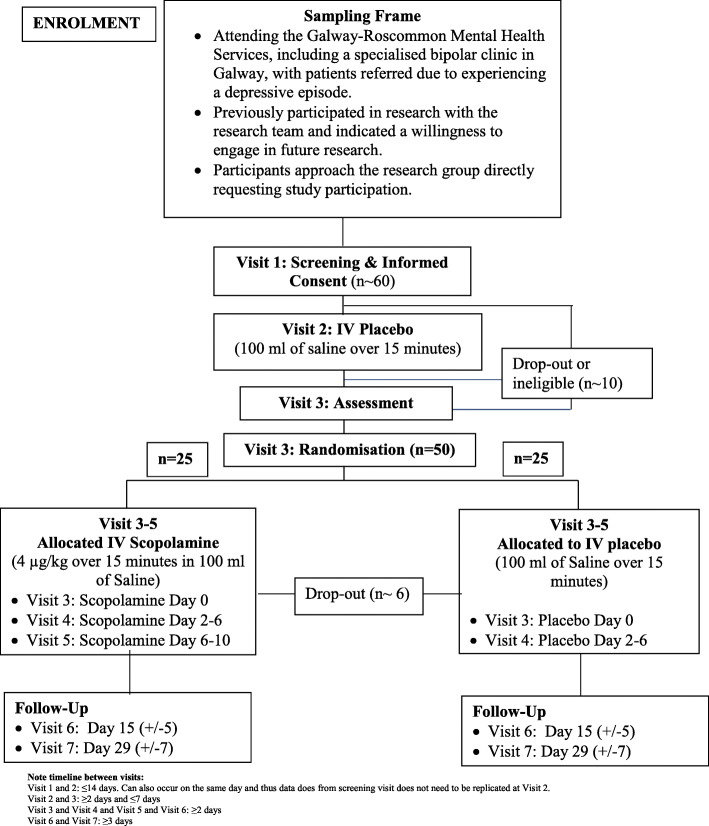
Study design. Note timeline between visits: Visits 1 and 2: ≤ 14 days. Can also occur on the same day and thus data from screening visit does not need to be replicated at visit 2. Visits 2 and 3: ≥ 2 days and ≤ 7 days. Visit 3 and visit 4 and visit 5 and visit 6: ≥ 2 days. Visit 6 and visit 7: ≥ 3 days

Participants will receive scopolamine or placebo in addition to their current treatment regimen. At least 2 days will elapse between IV infusions (visits 2–5). Two follow-up visits (visits 6 and 7) will occur at day 15 (± 5 days) and day 29 (± 7 days) with at least 2 days between visits 5 and the first follow-up visit (visit 6) and at least 3 days between the two follow-up visits (visits 6 and 7). If a trial participant requires medical intervention during or post-infusion, the treating physician must assess if it is appropriate to proceed with the current and/or future infusions. The flow of the study visits is presented in Fig. 1.

### Psychometric instruments

#### Clinical instruments

##### Alcohol Use Disorder Identification Test (AUDIT) [25]

A 10-item questionnaire to identify harmful use or dependence on alcohol.

##### Clinical Global Impression (CGI) [26]

A rating scale that measures illness severity (CGI-S) (1–7) and global improvement (CGI-I) (1–7) and has an efficacy index (which relates therapeutic effects to adverse effects) (1–4).

##### Fagerstrom Test for Nicotine Dependence [27]

A 6-item questionnaire examining nicotine dependence.

##### Global Assessment of Functioning (GAF) [28]

A rating scale from 1 to 100 that measures an individual’s functioning and is observer rated.

##### Hamilton Depression Rating Scale (HDRS) [29]

A 21-item observer rated instrument that measures symptoms of depression.

##### Montgomery and Asberg Depression Rating Scale (MADRS) [30]

A 10-item observer rated scale that measures symptoms of depression.

##### NEO Personality Inventory-Five Factor Inventory (NEO PI-FFI) [31]

This 60-item personality inventory measures personality traits in five dimensions (neuroticism, extraversion, openness to experience, agreeableness and conscientiousness).

##### Patient Rated Inventory of Side Effects (PRISE) [32]

A 9-item self-report instrument measuring adverse effects associated with the gastrointestinal, genitourinary, nervous and cardiac systems, sense organs, skin, sexual functioning, sleep and other general adverse effects.

##### Profile of Mood State (POMS) [33]

A rating scale where 65 adjectives are rated by participants on a 5-point Likert scale. Six factors are derived from these adjectives (tension, depression, anger, fatigue, vigour and confusion).

##### Structured Clinical Interview for DSM-5 Disorders (SCID-5-RV) [34]

This is a semi-structured interview that evaluates pathology and includes a demographic section followed by nine diagnostic modules, including two that examine mood disorders.

##### Visual Analog Scale (VAS)

A self-report scale used to indicate on a 10-point Likert how participants feel they are on various items, with the VAS utilised for the following terms in this study: (1) happy, (2) restless, (3) sad, (4) anxious, (5) anger, (6) drowsiness and (7) alertness. These items are consistent with those used by Furey et al. [10] in their study of scopolamine in mood disorders.

##### Young Mania Rating Scale (YMRS) [35]

An 11-item observer-rated instrument that measures symptoms of elation.

#### Cognitive Instruments

##### The Cambridge Neuropsychological Test Automated Battery (CANTAB) [36]

Tests conducted from this battery will entail the following: (1) Emotion Recognition Task (ERT) (assesses ability to identify emotions through facial expressions), (2) Paired Associates Learning (PAL) (assesses visual memory and new learning), (3) One Touch Stockings of Cambridge (OTC) (assess executive function through spatial planning and working memory), and (4) Rapid Visual Information Processing (RVP) (assesses attention).

##### Wechsler Adult Intelligence Scale 3^rd^ Edition (WAIS-III) [37]

Full scale, verbal and performance Intelligence Quotient (IQ) will be estimated using an abbreviated version of this instrument and will comprise the verbal subscales of vocabulary and similarities, and the performance subscales of block-design and matrix reasoning.

See Table 1 for the schedule of these psychometric assessments.

**Table 1:** Schedule of assessments

Procedures	Visit 1 Screening	Visit 2 PLACEBO			Visit 3 Randomisation Day 0 Scopolamine or placebo			Visit 4 Day 4 (± 2 days) Scopolamine or placebo			Visit 5 Day 8 (± 2 days) Scopolamine or placebo			Visit 6 Day 15 (± 5 days) Follow-Up	Visit 7 Day 29 (± 7 days) Follow-Up
		IV infusion			IV infusion			IV infusion			IV infusion				
		Pre	During	Post	Pre	During	Post	Pre	During	Post	Pre	During	Post		
Signed informed consent	X														
Inclusion/exclusion	X	X			X										
Randomisation					X										
IWRS					X			X			X				
Demography	X														
MEHI	X														
Medical/surgical history/history BPD	X														
Current medication	X	X			X			X			X			X	X
Vital signs—HR, BP, and RR	X	X		X	X		X	X		X	X		X		
SCID-RV	X														
HDRS	X	X			X			X			X			X	X
YMRS	X	X			X			X			X			X	X
AUDIT	X														
Pregnancy discussion	X	X			X			X			X				
Contraception advice	X	X			X			X			X				
Serum pregnancy test^Δ^		X													
Pregnancy urine dipstick^Δ^		X			X			X			X				
U&Es, LFTs, TFTs°	X	X													
Fagerstrom		X													
CGI-S		X			X			X			X			X	X
CGI-I					X			X			X			X	X
VAS		X		X	X		X	X		X	X		X		
MADRS		X			X			X			X			X	X
GAF					X						X			X	X
ECG		X													
Height (cm) and weight (kg)		X													
IV cannulation		X			X			X			X				
Infusion administration			X			X			X			X			
Adverse events		X	X	X	X	X	X	X	X	X	X	X	X	X	X
Cannulation site check		X		X	X		X	X		X	X		X		
POMS (optional)		X		X	X		X	X		X	X		X	X	X
CANTAB ^b^ (optional)				X			X							X	
WAIS ^a^ (optional)				X^a^			X^a^			X^a^			X^a^	X^a^	X^a^
NEO-PI-FFI ^a^ (optional)				X^a^			X^a^			X^a^			X^a^	X^a^	X^a^
PRISE (optional)				X			X			X			X		
Subjective assessment															X

^a^Can be undertaken at any of the marked visit

^b^These include the Emotion Recognition Task (ERT), Paired Associates Learning (PAL), One Touch Stockings of Cambridge (OTC) and Rapid Visual Information Processing (RVP) (assesses attention) tests

^Δ^Pregnancy test (serum pregnancy test and pregnancy urine dipstick), when required, for female participants only. Serum result confirmed prior to visit 3

°Blood tests for U&Es (urine and electrolytes), LFTs (Liver Function Tests) and TFTs (Thyroid Function Tests) should be confirmed within acceptable ranges in the previous 4 months of the screening (visit 1). Can be performed if required at visit 1 or 2 (results must be confirmed as acceptable prior to infusion)

*AUDIT*, Alcohol Use Disorder Identification Test; *BP*, blood pressure; *CANTAB*, Cambridge Neuropsychological Test Automated Battery; *CGI-I*, Clinical Global Impression—Improvement; *CGI-S*, Clinical Global Impression—Severity; *ECG*, electrocardiograph; *Fagerstrom*, Test for Nicotine Dependence; *GAF*, Global Assessment of Functioning; *HDRS*, Hamilton Depression Rating Scale; *HR*, heart rate; *IV*, intravenous; *IWRS*, Interactive Web Response System; *MADRS*, Montgomery and Asberg Depression Rating Scale; *MEHI*, Modified Edinburgh Handedness Inventory; *NEO PI-FFI*, NEO Personality Inventory-Five Factor Inventory; *POMS*, Profile of Mood States; *PRISE*, Patient Rated Inventory of Side Effects; *RR*, respiratory rate; *SCID-RV*, Structured Clinical Interview for DSM; *VAS*, Visual Analogue Scale; *YMRS*, Young Mania Rating Scale; *WAIS*, Wechsler Adult Intelligence Scale

### Data collection and randomisation

Clinical interviews and psychometric assessments will be collected by trained clinicians and research associates with a background in psychiatric research. All research staff involved in administration of clinical instruments or data-input will have up-to-date training in Good Clinical Practice (GCP) [38]. Infusions will be dispensed by the pharmacy and prepared by unblinded nurses, all attached to the CRFG.

Participants will be randomly assigned to receive either scopolamine or placebo in a 1:1 ratio. Randomly permuted blocks of sizes 4 and 6 will be used to ensure similar numbers of participants in each arm of the trial. Randomisation will be stratified by the HDRS score at trial entry (a score of < 23 indicating a mild-moderate depressive episode and a score ≥ 23 indicating a severe depressive episode). This will help ensure greater balance between arms in the final trial sample and increase the efficiency of our treatment effect estimates.

A validated randomisation system will be used at visit 3 (after the HDRS and YMRS are completed) to randomise patients to either arm. This centralised system will ensure allocation concealment, preventing blinded trial staff from knowing which treatment group will be allocated. Blocks of randomly varying length will also reduce the predictability of the allocation sequence. Randomisation will be carried out by the pharmacy team. In the case of an emergency, when knowledge of the treatment assignment is essential for the clinical management of the participant, a treating physician may request to unblind a single participant.

### Treatments

#### Treatment group

Participants randomised to the scopolamine group will receive a 15-min infusion of IV scopolamine in 100 ml saline, repeated over 3 visits (visits 3, 4, and 5).

#### Placebo group

Participants randomised to the placebo group will receive a 15-min infusion of IV saline, repeated over 3 visits (visits 3, 4, and 5).

Non-adherence will be extremely unlikely given the mode of administration, with participants monitored by a team researcher throughout the infusion period.

### Sample size calculations

Based on previous data [9], the allocation of 22 participants to each arm will ensure a power of ≥ 85% to detect a 50% reduction (treatment response) on the HDRS in the scopolamine compared to the placebo group. The sample size is based on a 2-sample *t*-test with a conservative standard deviation of 13 units and a significance level of 0.05. Allocating 25 participants to each arm will allow a loss to follow-up of approximately 12% post-randomisation (6 participants missing at visit 6 follow-up). It is suggested that approximately 60 participants will need to be recruited to ensure 50 participants (83.3%) are eligible for randomisation (visit 3). All participants will attain a placebo infusion at visit 2.

### Safety monitoring

Adverse events (AEs) and serious adverse events (SAEs) will be recorded on AE case report forms for all participants from the time of consent for the duration of their participation in the study. A full AE review will be conducted at each study visit to ensure that a complete evaluation of the safety and tolerability of the investigational medical product (placebo or scopolamine) is conducted. The site investigator will follow-up all AEs reported during the treatment period until resolved, considered stable, including completion of patient participation in the trial (i.e. final follow-up visit). All SAEs will be followed-up until resolution or until they are clearly determined to be due to a participant’s stable or chronic condition or intercurrent illness(es) including after trial completion if required. SAEs will be reported to the sponsor and principal investigator within 24 h after site awareness of the SAE to find a reasonable solution. Any suspected unexpected serious adverse reaction (SUSAR) that potentially occur will be reported by the sponsor to the competent authority (HPRA and/or EudraVigilance), the approving ethics committee and PI. Indemnity is in place pertaining to any adverse effects experienced by study participants.

### Statistics

#### Primary effect analysis

The mean scores at visit 6 will be compared across the study arms using an analysis of co-variance (ANCOVA) model. The response variable will be the change in HDRS score (primary outcome measure) from visit 3 to visit 6. For this effect analysis of the primary outcome, HDRS score at visit 3 is added as a co-variate as it is expected that pre-randomisation measurements of the HDRS score will be correlated with scores obtained at visit 6. The inclusion of stratifying variables (i.e. severe v. mild or moderate depressive symptoms at baseline) and other variables as covariates (i.e. bipolar I or II disorder) will be considered as appropriate to increase the power to detect significant differences between the groups. Inverse probability weighting will be applied to the primary outcome analysis. These weights will be derived based on the inverse of the probability of a patient’s data being missing given their pre-randomisation measurements. This will ensure the estimate and inference is more representative of all patients randomised, reducing bias in the estimation of the treatment effect due to participants being lost to follow-up and missing data. Inference regarding the treatment effectiveness will focus on the point estimate, confidence interval and p-value for hypothesis confirmation.

#### Secondary effect analysis

Secondary outcomes will be analysed as appropriate (according to the distribution of each outcome), comparing the difference between groups at the specified follow-up visit and relative to baseline values (where specified/measured). Baseline variables predictive of each outcome (the measure at baseline where available), and stratifying variables will be included in ANCOVA or generalised linear models of outcome variables. For secondary outcomes (i.e. acute and long-term effects on cognition (CANTAB), change in other mood rating scales including the MADRS and POMS, changes in functioning (GAF), treatment changes), the focus will be on the point estimates and confidence intervals for hypothesis generation.

Additional analysis of the primary outcome will compare the time course of HDRS under placebo and treatment using a mixed-effects model including fixed-effects terms for time since randomisation/study visit, trial arm and their interactions and a random effect to account for correlation of multiple observations per participant.

### Ethical approval

Required documents including the study protocol (V3.0, 18 December 2020), informed consent form, participant information leaflet and any other required documents (i.e. psychometric instruments) have been submitted and approved by the Galway University Hospital's Research Ethics Committee and the Health Products Regulatory Authority (HPRA). The sponsor (Research Office, NUI Galway) will submit and obtain approval from the above parties for substantial amendments to the original approved documents. The sponsor is responsible for implementing and maintaining quality assurance and quality control systems in line with written standard operating procedures (SOPS), ensuring data are recorded and reported in compliance with the protocol, Good Clinical Practice and regulatory requirements.

### Data management and monitoring

All study information will be stored in Microsoft Excel 365 by a data manager using an electronic database. The database will record all subject data to include the baseline characteristics, pre- and post-assessments, and possible adverse events. The computer and database will use password protection with the database only accessible to the study researchers. To ensure the confidentiality of the data, all subjects will be provided with identification numbers. All researchers will have access to the final trial data. Electronic data and paper documents will be kept for 5 years, after which they will be destroyed.

A Data Monitoring Committee (DMC), which is independent from the sponsor and competing interests, comprises clinical experts, trial experts, investigators and a statistician and will conduct regular monitoring tests and interim analyses (approximately every 4 months) to ensure the authenticity of the data. A trial steering committee, comprising the PI, clinicians and researchers with experience in this area, will meet every 6 months to review study progress and address any concerns subsequent to DMC reports. The study will be monitored on an approximate 6 monthly basis by the sponsor, entailing a blinded and unblinded study monitor of extensive experience. These monitoring visits will ensure appropriate patient eligibility for the study, staff training, accurate recording of data including source data verification and protocol deviations. The study will be open to external auditing by the HPRA (a process independent from investigators and the sponsor), throughout the time-frame of this study to ensure adherence to GCP.

The end of trial will be the date of the last visit of the last participant (50th randomised participant). The trial may be terminated prematurely if (1) new information about safety or efficacy becomes available (including from interim analysis of the DMC), (2) there is unsatisfactory progress of the study, including unsatisfactory progress in relation to participant recruitment or (3) deemed necessary by the sponsor, principal investigator, DMC, trial steering committee and/or the funder (Stanley Medical Research Institute).

This trial may be subject to internal or external auditing or inspection procedures to ensure adherence to GCP. Access to all trial-related documents will be given at that time.

### Dissemination

The results of this study will be published in peer-reviewed journals and be presented at domestic or international academic congresses. Individualised results will be reported by the researcher to the participants of this study, after data publication in peer-reviewed journals. Data will be available for public access via the National Institute for Mental Health Data Archive 1 year after submission of all participant data.

## Discussion

The purpose of this study is to examine the efficacy and safety of IV scopolamine compared to placebo in individuals with bipolar disorder who are experiencing a depressive episode. In addition, this study will also assess whether IV scopolamine, compared to placebo, impacts cognition and functioning in individuals with bipolar disorder experiencing a depressive episode of at least moderate severity. This is the first study to our knowledge to investigate the antidepressant effects of IV scopolamine in an exclusively bipolar disorder cohort. This study can help further research investigate the potential antidepressant effects of anticholinergic medications.

Some commonly documented adverse effects of IV scopolamine include drowsiness, blurred vision, dry mouth, thirst and urinary retention. No serious adverse events are expected from the IV scopolamine infusion, and no serious adverse events were witnessed in previous studies using IV scopolamine in depressed patients.

In relation to ethical considerations and patient confidentiality, the vulnerability of this study group is fully appreciated, and every effort will be undertaken to protect their safety and well-being. During their time on the trial, participants will continue usual care, as recommended by their responsible clinical team.

In line with the applicable regulatory requirements, consenting processes will be standardised and a robust standard operating procedure (SOP) for consenting participants will be adhered to. Patients will be given an information leaflet prior to participating in the study. The consenting process will be performed by trained clinical staff (principal investigator and sub-investigators with GCP certification) and will include providing a detailed explanation of the study, along with ample time for the research participants to ask questions about the study. Once agreeable, both the research participant and clinical staff taking consent will sign the consent form. A copy will be given to the participant and another copy will be stored in their medical file. In addition to having a full awareness of all study procedures prior to entering the study, participants will understand that research participation is completely voluntary and that they can withdraw from the study at any time.

## Trial status

Recruitment has commenced in March 2021. The duration of this study is 3 years, or until the number of necessary participants who have completed randomisation (*n* = 50) has been attained. It is consequently anticipated that participant recruitment will finish in March 2024.

## Sponsor

Vice-President for Research, National University of Ireland Galway

E-Mail: vpresearch@nuigalway.ie

Tel: +353-91-495678

## Data Availability

Data will be available for public access via the National Institute for Mental Health Data Archive (Bethesda, MD, USA) 1 year after submission for participant data where informed written consent is provided.

## Abbreviations

AE: Adverse event
ANCOVA: Analysis of covariance
AUDIT: Alcohol Use Disorder Identification Test
CANTAB: Cambridge Neuropsychological Test Automated Battery
CGI-I: Clinical Global Impression—Improvement
CGI-S: Clinical Global Impression—Severity
CRFG: Clinical Research Facility, Galway
DMC: Data Monitoring Committee
DSM: Diagnostic Statistics Manual
ERT: Emotion Recognition Task
GAF: Global Assessment of Functioning
GCP: Good Clinical Practice
HDRS: Hamilton Depression Rating Scale
HPRA: Health Products Regulatory Authority
IV: Intravenous
MADRS: Montgomery and Asberg Depression Scale
MDD: Major depressive disorder
NEO PI-FFI: NEO Personality Inventory-Five Factor Inventory
OTS: One Touch Stockings of Cambridge
PRISE: Patient Rated Inventory of Side Effects
PAL: Paired Associates Learning
POMS: Profile of Mood States
REC: Research Ethics Committee
RVP: Rapid Visual Information Processing
SAE: Serious adverse event
SCID-RV: Structured Clinical Interview for DSM—Research Version
SOP: Standard Operating Procedure
SUSAR: Suspected Unexpected Serious Adverse Reaction
VAS: Visual Analogue Scale
WAIS: Wechsler Adult Intelligence Scale
YMRS: Young Mania Rating Scale
